# Sail or sink: novel behavioural adaptations on water in aerially dispersing species

**DOI:** 10.1186/s12862-015-0402-5

**Published:** 2015-07-03

**Authors:** Morito Hayashi, Mohammed Bakkali, Alexander Hyde, Sara L. Goodacre

**Affiliations:** School of Biology, University of Nottingham, Nottingham, UK; Department of Zoology, The Natural History Museun, London, UK; Environmental Education Center, Miyagi University of Education, Miyagi, Japan; Departamento de Genetica, Facultad de Ciencias, Universidad de Granada, 18071 Granada, Spain; 63 High Street, Bonsall, Matlock, Derbyshire, UK

## Abstract

**Background:**

Long-distance dispersal events have the potential to shape species distributions and ecosystem diversity over large spatial scales, and to influence processes such as population persistence and the pace and scale of invasion. How such dispersal strategies have evolved and are maintained within species is, however, often unclear. We have studied long-distance dispersal in a range of pest-controlling terrestrial spiders that are important predators within agricultural ecosystems. These species persist in heterogeneous environments through their ability to re-colonise vacant habitat by repeated long-distance aerial dispersal (“ballooning”) using spun silk lines. Individuals are strictly terrestrial, are not thought to tolerate landing on water, and have no control over where they land once airborne. Their tendency to spread via aerial dispersal has thus been thought to be limited by the costs of encountering water, which is a frequent hazard in the landscape.

**Results:**

In our study we find that ballooning in a subset of individuals from two groups of widely-distributed and phylogenetically distinct terrestrial spiders (linyphiids and one tetragnathid) is associated with a hitherto undescribed ability of those same individuals to survive encounters with both fresh and marine water. Individuals that showed a high tendency to adopt ‘ballooning’ behaviour adopted elaborate postures to seemingly take advantage of the wind current whilst on the water surface.

**Conclusions:**

The ability of individuals capable of long-distance aerial dispersal to survive encounters with water allows them to disperse repeatedly, thereby increasing the pace and spatial scale over which they can spread and subsequently exert an influence on the ecosystems into which they migrate. The potential for genetic connectivity between populations, which can influence the rate of localized adaptation, thus exists over much larger geographic scales than previously thought. Newly available habitat may be particularly influenced given the degree of ecosystem disturbance that is known to follow new predator introductions.

## Background

Aerial dispersal is one of the influential dispersal mechanisms that allows movement across short to sometimes vast, even intercontinental, distances [1–4]. It is a key factor that shapes the spatial structure of populations within a species, and often a key determinant of the pace and scale of invasion of new areas. But how aerial dispersal has evolved and is maintained within individual species is still unclear [1–3, 5, 6].

One central and yet unanswered question is: what counterbalances the cost of dispersal [7–10]? In his book, Darwin expected that the ability of terrestrial organisms to survive encounters with water might decrease the risk of dispersal and promote terrestrial organisms to cross aquatic areas [11]. However, it has thus far been difficult to empirically connect dispersal cost and tendency [8–10].

Here we use a previously established method [12–14] to score long-distance aerial dispersal tendency in twenty-one common spider species (mainly linyphiids) exposed to suitable wind speeds in two different types of arena, on water and on a dry surface. We show that (i) many of these common spider species have individuals that can ‘sail’ on water using wind power alone (both in turbulent, still, fresh, and salt water conditions), and (ii) the ability to sail is tightly associated with tendency for airborne dispersal in this group of terrestrial spiders. We used spiders as a study organism because of their ecological importance as predators of a wide range of arthropods, including pest species, and because they are often the first colonizers of new areas with consequent implications for ecosystem development [15].

Spiders’ impact on ecosystems are potentially extremely large due to their efficient prey consumption as a top predator [16–18] and the possibility for significant numbers of invasions in a short span through airborne dispersal (termed ballooning) [12–14, 16–19]. Ballooning spiders are estimated to move a total of up to 30 km per day when wind conditions are suitable (each mean single flight is calculated as 500 m) but the distribution of dispersal distances is thought to be highly leptokurtic with many individuals moving smaller distances and a small proportion of individuals moving significantly further [19–21]. However, whilst able to control the decision to become airborne or not, ballooning individuals cannot predict where and how far they will travel in any one flight [22, 23]. Using ballooning as a dispersal strategy therefore involves taking a significant risk as, after each ballooning event, the airborne spider could end up landing in a habitat that is not suitable for its survival. Unsuitable habitats, understood here as sink habitat where spiders cannot survive for significant periods of time have been accepted to include water areas (*i.e.*, puddles, marshes, rivers, lakes, seas, oceans) that lie within a ballooning flight distance of the spider’s habitat [24].

Despite the apparent risks associated with ballooning, the trait appears to have been maintained throughout the spiders’ evolutionary history or to have evolved many times. Many spider species have worldwide distributions [23–26] and their aerial dispersal capability is thought to explain why they have been recorded as the first colonizers of new habitats —such as reclaimed lands [25, 26] and volcanic islands [24, 27, 28]. Spiders have also previously been observed and reported from the middle of oceans ([29–31]; reviewed in [24]).

Movement across water surfaces taking advantage of wind currents has been reported in pioneering work on species that have a particularly close association with water, such as *Dolomedes* raft spiders [32], but it has not been documented in strictly terrestrial species such as those used in the current study which are highly dispersive and known to use long-distance aerial dispersal throughout their life stages [24]. In our study we use laboratory experiments and observations to test whether common ballooning linyphiid and tetragnathid spiders, which respectively represent ~ 11 % and 2 % of all spider species ([33] http://www.wsc.nmbe.ch/), have evolved strategies that may allow them to survive on water.

## Results

**Fig. 1:**
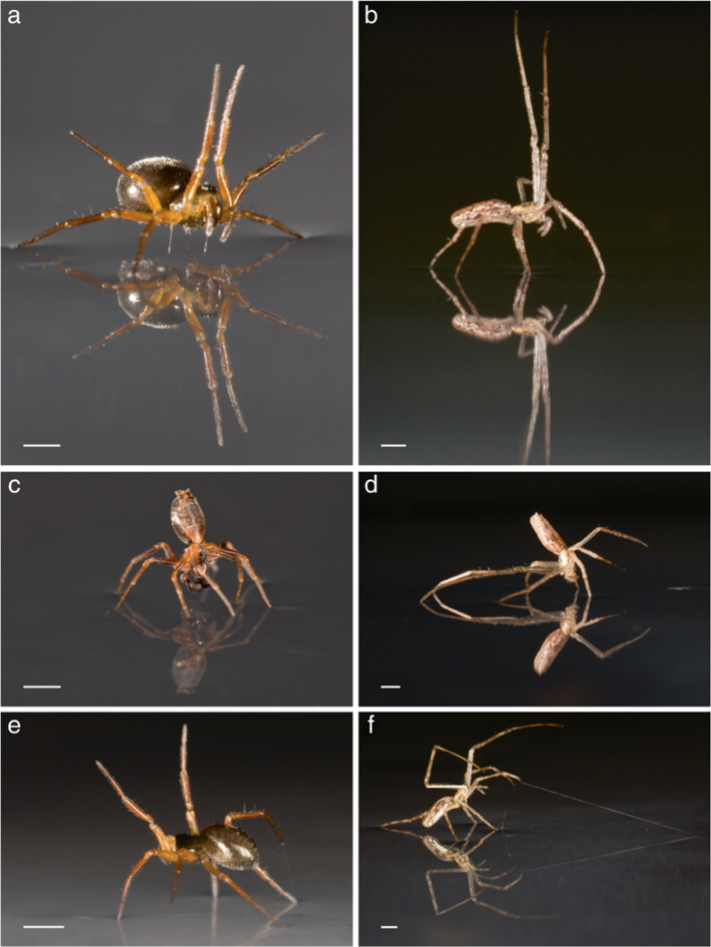
Spider behaviour on water surface. Sailing behaviour: linyphiid (**a, c**) and tetragnathid (**b, d**) spiders moving on the water surface with their legs (**a, b**) or abdomen (**c, d**) used as sails. When the abdomen was used the behaviour was referred to as upside-down sailing. A spider can sail stably even on turbulent sea salt water. Anchoring behaviour: use of silk as anchor to slow down or stop movement on water surface by linyphiids (which dropped the anchoring silk) (**e**) and the tetragnathid (which dragged the anchoring silk after it caught a floating object) (**f**). Each scale bar represents 1 mm

**Fig. 2:**
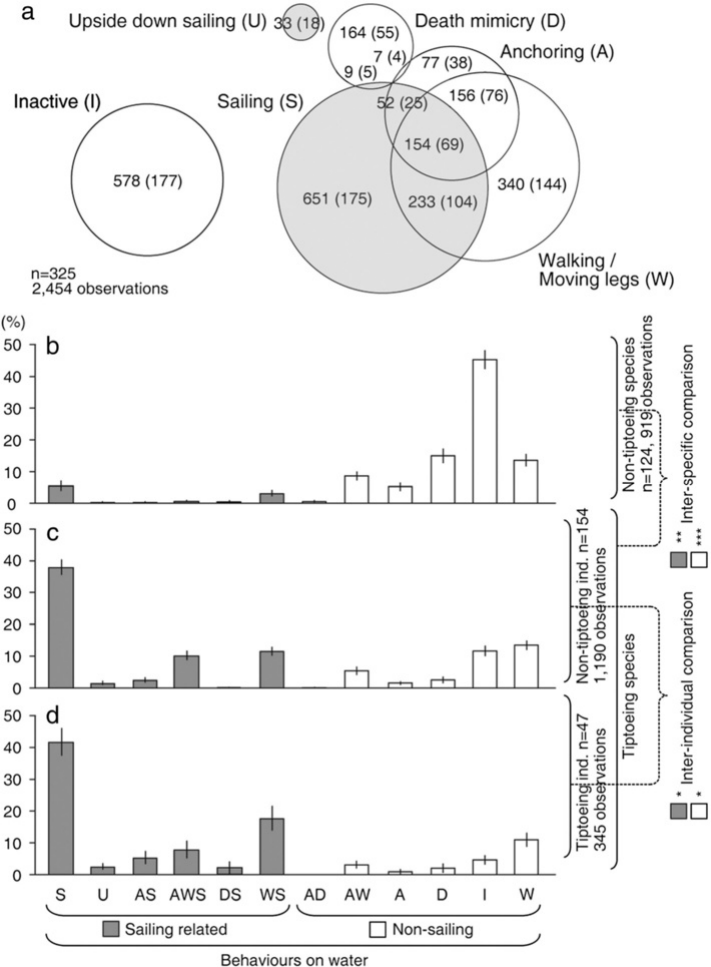
Spider behaviour on water surface and tiptoeing (ballooning) tendency. Behavioural categories and their prevalence (**a**). Overlap areas indicate combined behaviour. Numbers in parentheses indicate the number of individuals that performed each behaviour. Proportion of each behaviour in species where no individual showed tiptoeing (non-tiptoeing species) (**b**), in non-tiptoeing individuals belonging to species where tiptoeing was observed (**c**) and in tiptoeing individuals (**d**). The inter-species comparisons were between tiptoeing and non-tiptoeing species and the inter-individual comparisons were between tiptoeing and non-tiptoeing individuals, taking account of species difference. Asterisks indicate the level of statistical significance of the likelihood ratio test (*: *p* <0.05, **: *p* <0.005, ***: *p* <0.0005). Error bars, s. e. m

The behaviour of a total of 325 adult spiders belonging to 21 species was observed on water (Fig. 1). All the individuals tested had water repellent legs and we observed six single behaviours and six behavioural combinations as follows (Fig. 2). *Sailing (S)*: Once on the water surface, spiders react to the wind by raising their legs as sails (Fig. 1a, b). Sailing spiders smoothly and stealthily slide on the water surface without leaving any turbulence. *Upsidedown sailing (U)*: When on water, the spider reacts to the wind and raises its abdomen as a sail, in a handstand-like posture, and slides on water (Fig. 1c, d). *Anchoring (A)*: The spider releases silk on water surface and slows down its movement, or stops, against the prevailing wind (Fig. 1e). When the silken thread touches a floating object, the spider starts to walk on the silk until it reaches the floating object. *Walking/Moving legs (W)*: The spider attempts to walk on the water surface by rapidly propelling its legs and in the majority (~76 % of the time in our study), it moves in a downwind direction. *Death mimicry (D)*: The spider stays still and seems to mimic death on the water surface. Some individuals stopped moving for a few seconds then started to move again. We therefore used 60 s as the minimum time that a spider has to spend motion-less for its behaviour to be categorized as death mimicry. Death mimicry behaviour is likely to be a predator avoidance strategy, as is common to many animals (e.g. [34, 35]).

As a control, 271 individuals were subjected to the same wind force and experimental conditions but in the absence of water. This allowed observation of spider reactions to wind on dry surfaces and, thus, the identification of water surface-specific behaviours. A single individual briefly raised its two front legs on dry surface; the remainder either kept walking or bent their legs down so as to resist the wind, suggesting that sailing behaviour is almost exclusively associated with being on water.

**Fig. 3:**
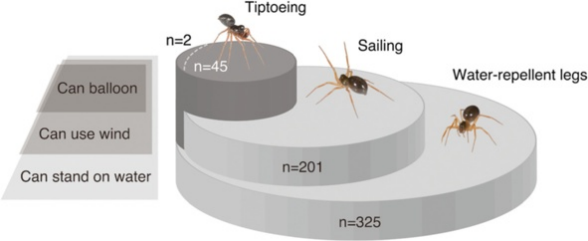
Water tolerance and tiptoeing. The relationship between tiptoeing, sailing and the ability to float on water. All tiptoeing individuals were also sailors, except for two individuals, suggesting that the sailing behaviour is almost completely associated with, and possibly a requirement for, the aeronautic behaviour

Spiders were also tested for their ballooning tendency on a dry surface. The aim was to test for a possible association between ballooning and sailing behaviours. We assessed this by studying tiptoeing, a pre-ballooning behaviour that is an indicator of the intent to balloon [12, 14]. We used a generalized linear mixed model in which the nested random factors take into account possible effects of pseudo-replication and sample bias. At the inter-species level, the likelihood ratio test showed that species with individuals that showed tiptoeing behaviour were more likely to exhibit sailing-related behaviours than species where tiptoeing was not observed (*X*^2^ = 9.969, *p* = 1.592 10^−3^ in sailing related behaviours and *X*^2^ = 12.82, *p* = 3.426 10^−4^ in non-sailing related behaviours, Figs. 2b and c-d). At the inter-individual level, those individuals that tiptoed were also more likely to sail than those that did not tiptoe (all tiptoeing individuals sailed with the exception of two; *X*^2^ = 5.406, *p* = 2.007 10^−2^ in sailing related behaviours and *X*^2^ = 4.413, *p* = 3.568 10^−2^ in non-sailing related behaviours, Fig. 2c and d). The association between the two behaviours may indicate that ballooners need to be able to sail (Fig. 3). In our study 7 out of the 21 species were categorized as ‘tiptoeing’ species even though the behaviour was observed in just a single individual. Exclusion of these species did not change the observed trends.

## Discussion

Our data indicate that, in contrast to the previously accepted view, long-distance dispersal of the spiders in our study is not limited by selection to avoid encounters with water because individuals display behavioural adaptations that allow them to survive encounters with aquatic environments. The sailing-related behaviours that we observe are specific responses to landing on water because individuals do not show this behaviour when experiencing similar conditions in the absence of water. Furthermore, ballooning and sailing-related behaviours appear linked such that individuals that balloon are the most eager ‘sailors’.

Sailing appears to be found in almost all of the individuals that aerially disperse but the reverse is not true (Fig. 3). Sailing behaviour in non-ballooning spiders is likely to increase survival near the wet areas and might also be useful to survive after rainfall, including flooding events. By releasing silk on water, sailing spiders seem to act like ships dropping their anchors to slow down or stop their movement. Our observations suggest that a possibility could be that the silk may sometimes work as a dragline for the water-trapped spider to attach to floating objects or to the shore. A spider that reaches a floating tree, for example, might be able to become airborne by ballooning from its surface, or from one of its non-submerged branches. The possibility of taking off directly from the water’s surface seems unlikely as, when exposed to wind currents on water, rather than flying, spiders appeared to ‘slide’ across the surface. In fact, none of our experimental spiders showed the typical pre-ballooning tiptoeing behaviour on water.

Our data indicate that ballooning is either a polymorphic or a polyphenic behaviour, since not all the individuals belonging to the ballooning species tested here showed the intention to balloon. Interestingly our study also points towards the occurrence of local adaptation since the individuals used in our study, which were taken from small islands within a nature reserve, showed less overall propensity to balloon than individuals taken from wider habitats, such as farm lands, where ballooning capabilities have been previously tested using similar experimental conditions and methods [14]. Given that we have demonstrated the potential for genetic connectivity amongst populations even when separated by water, these results imply that this localised selection is strong enough to counteract the effects of gene flow from adjacent populations.

Ballooning tendency is known to be population-dependent even in extreme aeronautic spider species [36–39]. Several environmental factors are known to influence spiders’ propensity to balloon, including thermal conditions during juvenile stages [37], the level of disturbance of the habitat [38], food availability to parents and their life stage [39]. Other factors, such as the size of the habitat patches and isolation level, are consistent with an underlying genetic basis for the observed variation amongst populations [36]. The isolation level may be particularly relevant to the current study given that spiders were collected from islands of less than 0.16 ha. Local adaptations that decrease windborne dispersal in small habitats, such as these islands, once expected by Darwin, are frequently reported in other species.

The linyphiids and single tetragnathid spider species used in this work are small bodied and do balloon as adults. Others, such as *Nephila pilipes* (Araneae: Nephilidae), are large bodied and known to balloon only as tiny spiderlings [40]. It is possible, therefore, that our results are not that far reaching and may apply to only small spiders. However, our bibliographical searches and calculations show that most ballooning spiders collecting by trapping in the wild [25, 26, 31] have water repellent legs [41]. Thus, a phenotype predicted to confer water tolerance is associated with ballooning in the vast majority of species characterized so far from at least 4 different regions (East China 99 %, *n* = 104; USA 86 %, *n* = 1,982; Switzerland 94 %, *n* = 4,268 and Australia >99 %, *n* = 503). The association between this phenotype and ballooning tendency is consistent with physiological adaptations resulting in water tolerance being an underlying requirement for the adoption and maintenance of the airborne LDD strategy (Fig. 3). This, together with the fact that all the spiders studied here had water repelling legs, might point to a widespread occurrence of water tolerance and ability of spider species to move across the water’s surface. Trichobothria, which are sensory hairs on the spiders’ legs, might play a part in sensing wind and water currents, but given that they are found in species where no ‘sailing’ behaviour was observed, their function seems unlikely to be solely related to the persistence of these behavioural strategies. The almost complete association between ballooning and sailing, seen in Figs. 2 and 3, might even suggest that sailing behaviour might probably even be a requirement of aeronautic behaviour. Whatever the case, it is interesting to note how a small movement from a tiny spider’s leg could allow better survival on water and might potentially have far reaching evolutionary and ecosystem-wide impacts because ballooning behaviour is widespread and prevalent within this ecologically important group of predatory arthropods.

## Conclusions

In conclusion, we discovered that a spider can smoothly move on water using its legs as sails and stop using its silk as anchor (Fig. 1). The propensity for sailing appears to be tightly linked to the tendency for aerial dispersal (Figs. 2 and 3), possibly because sailing alleviates the costs of landing on water. Spiders are often able to survive for long periods without food [42] thus water trapped spiders potentially persist for sufficient time to colonize otherwise out-of-reach, distant habitats. The aquatic environment thus appears to influence the persistence and effects of aerial dispersal with implications for the invasion of new and farther ecosystems throughout the evolutionary history of this group of species. Areas that are newly colonized by spiders may be particularly affected given the degree of ecosystem disturbance that is known to follow new predator introductions [43, 44].

## Methods

### Sample collection

We carefully hand-searched and collected spiders from 33 squared (1x1 m) quadrats on 18 islands of the Attenborough Nature Reserve and a similar quadrat at the Wollaton Park, Nottinghamshire, UK (species list in Table 1). All adult spiders found were included in our study. Our sample set comprised 325 adult spiders belonging to 20 linyphiid species and a single tetragnathid species used for experiments on water surface. To insure objectivity of the observer, species identification was carried out under a microscope only after all the experimentation and data collection were completed.

**Table 1:** Spider behaviour

Spicemen			Behavioural experiment on water				Behav. exp. on land	
Species	Family	Number of specimens	Sailing	Inactive	Other behaviours	Number of test	Tiptoeing	
							Yes	No
*Bathyphantes concolor*	Linyphiidae	25	4	81	111	196	0	25
*Bathyphantes gracilis*	Linyphiidae	17	10	55	71	136	0	17
*Dicymbium nigrum*	Linyphiidae	5	0	19	21	40	0	5
*Diplocephalus cristatus*	Linyphiidae	7	1	11	32	44	1	6
*Diplocephalus latifrons*	Linyphiidae	33	2	108	98	208	0	33
*Diplocephalus protuberans*	Linyphiidae	8	0	32	31	63	1	7
*Erigone atra*	Linyphiidae	30	40	29	131	200	7	23
*Erigone promiscua*	Linyphiidae	4	6	9	17	32	0	4
*Erigonidium graminicola*	Linyphiidae	38	138	19	143	300	14	24
*Gongylidium rufipes*	Linyphiidae	69	249	24	274	547	5	64
*Lepthyphantes alacris*	Linyphiidae	1	0	0	8	8	0	1
*Lepthyphantes zimmermanni*	Linyphiidae	6	8	3	36	47	1	5
*Linyphia montana*	Linyphiidae	3	0	7	17	24	0	3
*Monocephalus fuscipes*	Linyphiidae	27	0	132	79	211	0	27
*Oedothorax apicatus*	Linyphiidae	1	4	0	0	4	0	1
*Oedothorax fuscus*	Linyphiidae	4	16	0	16	32	0	4
*Oedothorax gibbosus*	Linyphiidae	1	4	0	0	4	0	1
*Troxochrus scabriculus*	Linyphiidae	1	0	3	5	8	0	1
*Erigone dentipalpis*	Linyphiidae	1	0	8	0	8	0	1
*Erigonella hiemalis*	Linyphiidae	1	0	8	0	8	0	1
*Tetragnatha extensa*	Tetragnathidae	43	169	30	135	334	18	25
Total		325	651	578	1,225	2,454	47	278

Table showing the total number of individuals included in our study belonging to each species with (i) the results of the behavioural tests on water. Each spider was tested up to eight times. The total number of trials during which different behaviours (e.g. sailing) were observed on water is shown and (ii) the results of the behavioural test on land. Each spider was tested once, for a total of 1 min

### Experiments for testing tiptoeing behaviour

Tiptoeing is a pre-ballooning behaviour that is widely recognized as unequivocal indicator of ballooning intention [12–14]. Tiptoeing behaviour was tested and scored for each individual spider that was separately exposed to wind in a 10 by 10 cm dry experimental arena surrounded by water. The observation lasted for one minute and the spider’s behaviour was recorded using the program Etholog [45]. Species and individuals were then classed as either tiptoeing (i.e., getting ready to balloon) or non-tiptoeing (showing no sign of getting ready to balloon).

### Preliminary experiments on categorizing aquatic behaviours

Behaviours on water were observed and categorized under wind speeds ranging from 2.85 to 78.0 cm/s. The following behaviours were scored: (S) sailing with legs, (U) sailing with abdomen (upside-down sailing), (W) walking or moving legs, (A) anchoring, (D) death mimicry, (I) no behavioural changes (inactive), and another six combinations of these (Fig. 2a). A more detailed description of each behaviour can be found in the results section.

### Behavioural experiments on water surface

The behaviours of spiders exposed to pump-generated air (i.e., wind) of 2.85 cm/s were observed between start and goal lines separated by 30 cm, in a shallow tray (600 by 380 by 18 mm) filled with water to 1 cm height. We used the lower wind speed rather than stronger wind in order to observe each spider behaviour for longer time periods and more accurately. For reproducibility, and to avoid possible bias from uncontrolled air currents in the laboratory, each experiment was carried out twice and in opposite directions (eight times in total). Experiments were carried out using both fresh and 3.5% sea salt water, and with and without turbulence. Turbulence was generated at one side of the experimental arena (tray) and the waves were ~0.5 mm height and had a periodicity of two waves per second. As a control, air was also directed at 271 of the 325 spiders that were used in our experiment on aquatic behaviours, but this time on a dried experimental arena. This allowed us to test whether the previously observed behaviours are water specific or not.

The fact that all our spiders did float on water and that their legs produced little dimples on the water surface (Fig. 1) suggested that they all have water repellent legs.

### Statistical analyses

A likelihood ratio test was used to estimate the relationships between behaviours on water surface (sailing versus not sailing) and ballooning (tiptoeing versus not tiptoeing) at both the species and individual levels. Two generalized linear mixed models (GLMMs) were compared, a model in which the explaining variable is the behaviours included S or U (sailing) or not and a null model in which the explaining variable is null. For the explaining variable to significantly explain the dependent variable in the model, the likelihood ratio test that compares the model and the null models must show a less than 0.05 p-value. To fit the binominal distributions of the data from the behavioural experiments on water (i.e. whether a sailing occurs or not) to the model, logit-link was selected in the GLMM. The binary tiptoeing data (tiptoeing versus not tiptoeing) were treated as a covariate. Species, individuals and measurement replicates were treated as nested random factors. Hence, we analyzed the relationship between the data on the tiptoeing behaviour and the sailing behaviour. All statistical analyses were performed using the software ‘R’ [46]. Analysis using GLMMs [47] were carried out using the program lmer in the lme 4 package [48] within ‘R’.
