# Erratum to: Sail or sink: novel behavioural adaptations on water in aerially dispersing species

**DOI:** 10.1186/s12862-015-0500-4

**Published:** 2015-10-20

**Authors:** Morito Hayashi, Mohammed Bakkali, Alexander Hyde, Sara L. Goodacre

**Affiliations:** School of Biology, University of Nottingham, Nottingham, UK; Department of Zoology, The Natural History Museun, London, UK; Environmental Education Center, Miyagi University of Education, Miyagi, Japan; Departamento de Genetica, Facultad de Ciencias, Universidad de Granada, Granada, 18071 Spain; 63 High Street, Bonsall, Matlock, Derbyshire, UK

## Erratum

The original version of this article [1] unfortunately contained a mistake. The funders information (RCUK) that should have been present in the Acknowledgments section was missing in both the HTML and PDF versions of this manuscript. The corrected Acknowledgements section is presented below with the additional information present in bold:

We thank F. Perfectti and J. M. Gómez at the Universidad de Granada, M. Kawata and S. Chiba at the Tohoku University, J. Brookfield and the late B. Clarke, FRS at the University of Nottingham for critical review of the manuscript; T. Miyashita at the Tokyo University, M. Schilthuizen at the Naturalis Biodiversity Center, Leiden and R. G. Gillespie at the University of California, Berkeley for fruitful discussions; G. Bowden and T. Sexton for assistance with sample collection; J. Ellis, C. Martin, S. Aitkin, J. Black, A. Hindmarsh and the late K. Corbett from the Attenborough Nature Reserve for helping to coordinate fieldwork; the Nottinghamshire Wildlife Trust and CEMEX for permission to collect samples. This work was supported by research fellowships from the Japan Society for the Promotion of Science and Daiwa Foundation, Japan. **SLG was supported by an RCUK Fellowship awarded to Professor Grierson, and by the School of Life Sciences at the University of Nottingham.** M. Bakkali wishes to thank the Spanish Ministerio de Ciencia y Tecnología and Ministerio de Ciencia e Innovación for the BFU2010-16438 and the Ramón y Cajal fellowship.
